# Foreign

**Published:** 1840-07-11

**Authors:** 


### FOREIGN.

*Contributions to Pathology—Cases of Hydrophobia, or Rabies.* By Alexander Kilgour, M. D.

No cases of hydrophobia had occurred in Aberdeen, or the north-east of Scotland, in the memory of any inhabitant until the present year, (1839,) when the following cases of it made their appearance. In the month of March considerable alarm was created by a bitch that was running about through the streets, and bit a considerable number of persons; but it was ascertained that the animal had been deprived of her puppies, and her ferocity was ascribed to this as its cause. She was destroyed; but in consequence of the excitement produced, the magistrates directed that no dog should be allowed to appear on the streets without being muzzled; and those found without the muzzle were taken to the watch-house and destroyed, or their owners fined. The muzzles used were leather straps, a part of which goes round the head of the dog, an inch or two above its nose, and prevents its opening its mouth to half its usual extent, or protruding its tongue. To save the expense of a muzzle made by a saddler, many of the poor people had their dogs' heads confined by pieces of cord or tape fastened tightly round them. Those not muzzled were confined at home, being generally tied up; or were led about by their owners on the streets with a string attached to them. In consequence of the alarm, and from the measures of security which were enforced, dogs were shunned, and when met with loose, they were hunted by boys until they were irritated, and then being declared mad, they were pursued until destroyed, or got clear of their pursuers. Whether from this system of hunting and baiting them, or that they really were diseased, and had, as usual when affected with rabies, run themselves to death in obscure and unfrequented places, there can be no doubt that several dogs were found dead within a mile or two of the town, and of these some had previously been seen running wildly about the country. Many rumours went abroad of strange dogs having been observed affected with disease, and of their biting others; but nothing could be gathered of a definite nature, when these reports were investigated.

In the three cases following, the history of the bite is given as far as could be ascertained. The first case, as well as the third, proved fatal at the patients' own houses; the other was admitted into the Infirmary, under the charge of my colleague, Dr. Dyce, who has furnished me with the report. I leave the third case to speak for itself. I did not see the patient in life, but there is no doubt on my mind, nor I believe on that of the medical gentleman who attended her, that it was a decided case of hydrophobia.

I have nothing to offer in regard to the pathology of the disease or its treatment. I believe we are as far as ever from having any satisfactory understanding of the one, as we are from possessing means of ensuring a successful issue in the other. There are two points, however, common to two of these cases, the second and the third, that they took place in individuals of a particularly nervous temperament; and that the most careful *post mortem* examination in the first case, and in the second to the same extent, except that the spine was not opened, discovered to us absolutely nothing that we could connect with the previous symptoms. Could any thing be gathered, in a physiological view, from the fact that all these patients, when pressed to take fluids, demanded a spoon with which to convey them to the mouth; and that they preferred taking substances in the state of pap, for example, porridge and milk, and bread and milk, or tea with biscuit soaked in it, to either solids or fluids? In Dr. Dyce's patient I observed, on my carrying a small white earthenware vessel with water in it towards the patient's bed, that he began to move his mouth and throat before I offered it, as if in the process already of deglutition, or rather mastication, and what he took from me was in small mouthfuls, and swallowed as if after mastication, instead of a steady draught.

Case 1. Thomas Alison, aged ten, residing at Footdee, of a healthy appearance, and described by a most intelligent person in whose vicinity he resided, and who daily saw him, as a remarkably smart and clever, but tricky boy, was bit on Friday the 10th of May by a dog. The dog had at one time been domesticated in the same neighbourhood, but had afterwards been taken into the country for a few miles, where he had been bitten by another strange dog, and on getting back to town on the day mentioned, had visited his old quarter. There was no reason for supposing at the time that he was diseased, and he was not previously considered vicious. A man in the building-yard, which he entered, was patting him, and the boy Alison put down his hand for the same purpose, when the dog bit him in the wrist.

The boy's mother stated that he was much alarmed from the bite, and that, since that time, he has been often disturbed in his sleep, rather fretful, and not as usual among the rest of the children. She washed the wound with spirits, and applied poultices. On the 21st of May, he went into the harbour in a boat, and losing his oar, became very much excited, and screamed until some persons went out to his assistance, and brought him ashore. But, residing close to the harbour, he was much accustomed to boating in it, and his mother stated that this situation would formerly have been in no way alarming to him. On Wednesday the 29th of May, she observed that he was unusually dull and inclined to sit by the fire. She thought his stomach was disordered, and gave him of her own accord a calomel powder, and on the following morning a dose of senna infusion. On this day, Thursday the 30th, he was first observed to have spasms. On Friday, at seven in the morning, Dr. Cuddie was sent for, and informed that the boy had hydrophobia. On visiting him, he was satisfied that such was the case; and he bled him and gave him some purgative medicine, and after that calomel and opium. At a few minutes past one of the same day, I saw him along with Dr. Cuddie and Dr. Ogston.

The instant we entered the room, and passed the end of the bed, which was close to the door, but covered, the boy started up with a scream, and placed himself in a sitting position in the farthest corner of it. My impression at that instant, and all the time I saw him, was, that I had never seen any thing like his appearance, but that of a child who had been greatly frightened, and not recovered from the fright. His countenance was anxious and unsettled, but not flushed or cadaveric. The predominant expression was that of extreme fright and terror. His eyes had a peculiarly unsettled but not wild expression. In a few seconds he was reconciled to our presence, but frequently sprung up to the sitting posture with a scream, and put his hand to his throat, as if he were choking, at the same time throwing his head up, as if he wished to inhale deeply. He would then handle or move the bed-clothes, but without any apparent determinate object; then sigh frequently and deeply, and become quieter. In a few miuutes again, the same was repeated. Sometimes he raised himself more than half out of bed, but raised his chin as if to lengthen his neck, and carried his hand to the throat and the breast. At these times there was an evident catch in the breathing, or difficult inspiration. There was in the intervals of these spasms of the throat, jactitation of the body, and twisting of the mouth, particularly drawing down of the angle of the mouth on the same side as the bite. He very frequently gave something between a sigh and a moan; he retched occasionally, and brought up a frothy fluid like saliva, which was adhesive and stringy. Whatever he did was in a hasty manner; and his question, "*Fat*," Aberdonicè for *what*, when he did not understand us, was given in a startled way,—not that he was frightened at us, but as if under fright from some other cause. On offering to touch him, he started up to the further corner of the bed; but, in an instant, on telling him that we wanted to feel his arm, he put it out to us. The same took place on offering to touch any part of his body; but on coaxing him a little, he took my hand, and conveyed it himself fiist to his left arm-pit, and then to the back of his neck; but he had the greatest sensitiveness in regard to touch, and no deliberate examination of any part could be made from his restlessness and uneasiness. When questions were put to him, he spoke sensibly, but hurriedly, and always as if in alarm. He appeared to take no interest in the conversation we had with his mother as to the history of the case, nor to regard our presence or look much at us, the frequent paroxysms keeping him in a constant state of alarm and pain. There was no matter or inflammatory redness about the scars on the wrist. His pulse, as far as could be ascertained, was very weak and rapid, about 120; the skin was cool; he had made urine, and his bowels had been fully opened; he had vomited (he told Dr. Cuddie) all his medicine. His mother stated that he had had a swelling in his arm-pit two or three days before, but, as far as he allowed examination, nothing of the kind could be felt. The blood taken in the morning was in a dark clot, and so soft that the probe could not raise it, but passed through it. A tin tumbler of water was offered him, which he rejected with a start; but on again coaxing him, he agreed to take a drink provided he got a spoon and a plate. He then took two or three spoonfuls of the water into a plate, and took a spoonful of the fluid from the latter, which he swallowed. His hands shook much, and he darted the spoonful to his mouth suddenly, after having, as it were, made up his mind to swallow the water, but immediately after, retching and vomiting took place.

Before we left him I poured some water from a height on the plate without his seeing me, and he instantly projected his hands in a supplicating attitude, with a countenance of extreme terror, and then burried himself under the bed-clothes. Dr. Cuddie stated the symptoms to have been the same in the morning, but that the pulse was stronger and the spasms more frequent. In the evening, at half-past seven, he was taking some porridge and milk; but his pulse was scarcely to be felt; the skin was cold, and his face was congested. He was also somewhat delirious, and inclined to be talkative, and rather witty with Dr. Cuddie, whom he best knew. He died about 11, in one of the paroxysms, according to his mother's statement.

The body was examined by the same parties, and Mr. Fraser, surgeon, nineteen hours after death.

The throat, chest, abdomen, head, and spine were most carefully examined, but nothing particularly deserving notice observed. The tongue was indented on the sides by the teeth, and there was some adhesive mucus on the palate. The glottis and pharynx were natural. The spaces betwixt the rings of the trachea were somewhat vascular. The œsophagus was pale as usual, except close to the cardiac opening, where the tint was pinkish. The mucous coat of the stomach was marked by some spots composed of close red dots. The intestines, particularly the colon, were much distended with air, and giving out the smell of turpentine, of which he had an enema.

The lungs were rather congested. The heart contained semi-fluid blood in both ventricles. The liver was natural.

The kidneys were rather vascular. The brain was natural; or if any change, it was that the great commissure and *corpora striata* were rather soft. Bubbles of air were observed in the veins; and not half an ounce of fluid in the brain. Some fluid was found in the theca of the spinal canal. The chord was in some places rather more vascular than usual; but as to this all were not of one mind.

The body was pale; the lips rather blue; the joints stiff. The largest scar was close to the wrist, on its internal and anterior side. It was jagged and irregular on the edge. On laying open the part, it was found to be placed over the ligament binding down the tendons, and immediately over the ulnar nerve, but not penetrating to it. There was no purulent matter, nor signs of inflammatory action. The other scar was on the back of the metacarpal bone of the thumb, but was healed up, or left only a slight mark. It was found immediately over the posterior branch of the muscular spiral, but there were no appearances of the bite having gone through the cellular tissue. The nerves were perfectly healthy, and no appearance of any inflammation of the absorbents in the arms was recognized. There was no swelling in the arm-pit.

Case 2. G. G., aged 28, married, a stout, muscular farm-servant, was admitted into St. Nicholas' Ward, about 2, P. M., on Monday the 12th of August, 1839, with symptoms of hydrophobia.

He stated that on the 7th of July, on attempting to tie up a dog, which was supposed to be unwell, he was bitten in the right forearm, near the wrist, and in the fore and upper part of the right thigh. To both wounds caustic was freely applied soon after the injury, and they were then dressed with basilicon and poultices until they healed, which took place in about a fortnight. He then remained free from complaint, excepting a degree of weakness, until the 7th of August, (five days ago,) when he felt occasional sharp pains in the fingers and right arm, extending towards the *axilla*, with numbness and diminution of power in the whole extremity. He continued at his work, however, until Saturday the 10th, when the pain in the arm increased, and extended to his neck. He then applied for medical aid. He was ordered a dose of calomel and jalap, with a mixture containing Mist. Camphoræ, ℥iii., Solut. Muriat. Morph. ʒi. M., of which he was to take a table-spoonful occasionally.

On Sunday, the 11th, he was restless from oppression about the chest, with some sense of choking. He had not taken the mixture as prescribed; the powder had operated well; the pain in the arm was less severe, but the arm was still numbed. His appetite was good, and he ate on this night a hearty supper before going to bed. He had not been asleep long when he was awoke by an oppressive sense of choking, from a feeling as if a lump lay at his chest, with increased pain in the right arm, and which he distinctly described as extending to his throat and chest, with almost total loss of power of the extremity. When he got up to drink some toast and water to quench his thirst, which was very urgent, he found he could not swallow it; and when he again tried and succeeded, the effort was followed by so severe a paroxysm of agitation, choking, and perfuse perspiration, as, to use his own words, "almost destroyed him." He slept none after this, and continued restless.

When first seen on admission into the Hospital on the 12th, he was being fed by the nurse (out of a white vessel,) with bread and milk, of which he was reported to have swallowed, though with difficulty, some spoonfuls. He complained much of want of sleep; was tranquil in manner; occasionally sighed deeply, but without irregular breathing; said the cold air annoyed him, and requested the door to be kept shut, and then gave the foregoing account of his complaint.

He expressed no disinclination to take fluids when offered him; but it was evident the resolution produced anxiety; and when the vessel was brought near him, his eye glistened, and his frame and manner became agitated and flurried, but without actual spasm. The impression on my mind at the moment was an unwilling compliance, or wished for evasion of the question, as he entreated in rapid succession not to be hurried; to give it in a spoon; and that it should be warm. Warm fluids produced less uneasiness than cold, and solids less than either. The cicatrices presented nothing unusual; they were very slightly tender, of a deep purple colour, without induration, and partially covered with a loosely adherent scale. Both arms were numbed, particularly the right or bitten arm, with which he is unable to lift any thing. He could, however, raise it to his head when requested. He had no numbness or uneasiness in the lower extremities. The pulse was about 100; tongue covered with a thick, tenacious, white coating, but moist; the body was covered with perspiration.

℞. Camphoræ; Calomelanos $\bar{a}$ gr. iii.; Opii Pulv. gr. i. M. Ft. pilul. ii. statim sumendæ.

8, P. M. He had several short sleeps. From the first he awoke agitated and alarmed, from the others quietly. He took some warm bread and milk with a little difficulty, but shrunk from cold water. He thought himself not so well. Had frequent eructations during the visit, which, each time, was followed by a slight tremor and uneasy motion of his body. On requesting him to drink, he was thrown into violent agitation before it had approached him, attended with catching and deep hurried inspirations, and he begged us to desist. Shortly after this he swallowed one or two spoonfuls hastily, and with much difficulty, perspiration continuing profuse. The tongue and pulse were much as at last report. He was ordered two pills containing Calomel. gr. iv., Opii gr. ii., Camphor. gr. iii., every four hours, and the following liniment to his back and breast: Tinct. Opii ℥i., Spt. Camph. ℥ss., Aq. Ammon. ʒii. M. A turpentine enema was directed to be given.

August 13, 9 A. M. Had taken six of the pills ordered last night, and he had slept several times quietly though shortly until 5 A. M., when he took the last pills. Soon after this he was attacked with violent spasmodic retching, with a copious flow of glutinous frothy saliva from the mouth, mixed with dark streaks, apparently blood. He complains much of a disagreeable taste in mouth, with a sense of burning at epigastrium and throat. The breathing not so hurried or oppressed, neither has he the same dread of liquids, having, he says, helped himself to some cold water. A pill of croton oil and crumb of bread, ordered at 6 A. M., has only now been taken, after much persuasion and increased difficulty. Feels inclined to eat, but positively refuses to take more medicine. The enema produced no effect. Pulse about 120; tongue more furred; sweating still profuse.

Noon. He is reported to have taken some biscuit soaked in tea, with little difficulty, and continued quiet and tolerably comfortable until half past 11, when the retching and flow of saliva returned. Has talked at times incoherently, affirming that he has been poisoned, and all around him have conspired to murder him. At present he is quiet, but his expression of countenance is more anxious; spoke sensibly; and, when requested, took a spoonful of water with less apparent reluctance and difficulty than last night; pulse 130. He was directed to have immediately a tea-spoonful of laudanum, to be repeated every third hour, and a purgative enema. About an hour after this, whilst I was visiting the other patients in the ward adjoining his closet, he became violently agitated, threw himself about in bed, talking loudly and incoherently, while the frothy saliva flowed copiously from his mouth, but without retching. The perspiration run from his face. This attack appeared to have been brought on by an occurrence in the ward,—a patient having suddenly expired, whose last shriek he appeared to associate with the cry of his child, about whom and his wife he raved. The paroxysm soon passed off, and he became submissive, lay with his eyes half closed, and swallowed a tea-spoonful of laudanum with little persuasion or difficulty. In the afternoon had several similar paroxysms of agitation. The laudanum was repeated, but was immediately rejected. The pills and enema he positively refused. About 6 P. M. he vomited a considerable quantity of a blackish fluid, resembling coffee-grounds, after which he lay in a state of collapse for about five minutes, when the whole body, (right arm excepted, which was uncovered, and hanging over the side of the bed,) became cold, and covered with a clammy sweat. About 7 P. M. the same symptoms returned, with a similar termination, and again about twenty-five minutes to 8 P. M., when, at the close of the paroxysm, he ceased to breathe. In the intervals between the vomiting, he was at times composed, and spoke sensibly, but more generally raved on the old theme of being poisoned; but during the whole time he knew and called those around by their names.

An inspection was not granted by his friends.

Case 3. 2d October, 1839. Mrs. D——, aged 52, married, the mother of a family, and of sober and regular habits, but of a nervous temperament and easily excited, had been complaining of what she thought was a cold since the afternoon of Thursday, the 26th of September, on which day she received a fright from a cat destroying a favourite bird of her son's. On Saturday, the 28th, she was out at market; and on that day she told her family that she was dying, and requested them to send for medical advice, to save reflections.

When visited on Sunday at 4 o'clock, she was found complaining of difficulty of swallowing, especially fluids, attended with occasional fits of dyspnœa, and inability of remaining at rest in bed. On offering her any thing, she felt a great sense of choking and suffocation, and she snapt at what was offered her, or took it in a hurried and jerky manner. She had a great terror at liquids, although she complained of much thirst. Her pulse was not quick, but weak; her mouth clammy.

On Monday, the 30th, she was in much the same state; slept very little, and when she did so, always awoke with a sense of suffocation. She preferred sitting up by the fire to being in bed. Bowels opened by laxative medicines. Had been ordered an anti-spasmodic mixture.

Tuesday, 1st of October. Spasms and excitement worse, but with difficulty had swallowed a little biscuit and tea. Was continually talking, but answered questions distinctly. Fancied she saw persons about her; had no sleep; had the same horror when any thing was put to her mouth, and snapt at it as before. At one o'clock she was in a convulsive fit, with twitchings of eyelids and cheeks, and mouth open, with the tongue partially protruded. She died at three o'clock, forty-seven hours from the period she was first visited.

The body was examined twenty-one hours after death. In the brain every part was natural, except that the left thalamus was internally of a slightly pinkish hue, and the consistence not, on the whole, so firm as the other one. The *velum interpositum* was more than usually vascular, and this state was very remarkable in the situation of the pineal gland. The veins in the *pia mater*, where it enters at the fissure of Sylvius, were very tortuous and large, but empty.

The viscera of the chest were natural. The only thing deserving notice in the abdomen was, that the stomach was remarkably small. Its mucous membrane, and that of the œsophagus, were quite healthy.

On considering all that we had observed in this inspection, and taking the symptoms, a few of which had been mentioned to me by the medical gentleman who attended her, in our walking to the house, (the above history having been furnished by him the day after,) I was struck much with the case. From her peculiar temper it had been looked upon as a nervous affection, or a case of hysteria; but it was stated by the friends, that the woman herself was acutely sensible of her horror at liquids, and that she had repeatedly told them she could not take them, but would make the attempt, and that the manner she did so was such as to astonish them all. She had also complained much of the cold air when moving the bedclothes about her; but they had not observed any thing particular in her appearance, nor heard her make any complaint on the door being opened. The husband also stated that she had retched often, and that what she brought up was froth and "like soap-suds."

I had observed a little dog on entering the passage of the house, and had asked them if the dog was theirs. The husband said that they had had a dog, but that it died about six weeks ago. They could not say what was the matter with it, but it drooped, and could not eat or drink, from its tongue being protruded, and so swelled that it could not get it back into its mouth. It was not vicious, and never had bit any one, nor been bit. In its illness it paddled with its mouth in water put down to it, but could not lap it up from the state of its tongue. It slavered much. The woman was extremely fond of it, and nursed it on her knee frequently. I examined her hands, but saw no scratches or sores. On further inquiries being made of the husband and sons two days afterwards,—the woman having been burried,—they stated that the dog had come home some time before its death, with a cut in its head, but whether by a dog or not they could not say; and they also stated that the deceased had a sore on her arm, which she was in the practice of making the dog lick, in the belief that the doing so would heal the sore.

It was apparent that the friends believed the woman's death was connected with the dog; but they wished it kept quiet, and it was with considerable reluctance they gave the preceding statement.—*Edinburgh Medical and Surgical Journal.*

---

*Case of Spontaneous or Idiopathic Emphysema.* By James Mouat, M. D., 13th Light Dragoons, Madras Service.—The evolution or secretion of air from the blood into the cellular tissue, is a very rare phenomenon. Indeed, so few cases are there detailed by medical authors, that I am induced to record a recent instance of spontaneous or intrinsic emphysema occurring in India.

Private William Preston, H. M. 13th Light Dragoons, aged 32½, in India seven years, a tailor by trade, dark hair, spare make, swarthy but healthy, was in hospital with symptoms of *dysenteria ac.* 4th July, and 14th August; and *hepatitis acuta*, 17th August, 1837; since which period he has been at his duty, and working in the tailor's shop till the evening of the 9th June, 1839, when he was admitted with pain and oppression in chest, and slight cough, attended by severe headach; heat of skin, succeeded at times by cold chills; much thirst; pulse quick and hard; tongue foul and loaded; bowels regular. He had been ill two days previous to his admission, and can assign no cause for his ailment.

He was immediately bled to thirty ounces, and suitably treated with purgatives, the *Solut. Antimon. Tartarizat.*, &c., with scarcely any relief. There being considerable fever the following morning, the bloodletting was repeated to twenty ounces. In the evening he still complained of much pain in the chest, with some dyspnœa, and quick respiration; pulse one hundred; much thirst. The bowels had been freely evacuated. Thirty leeches were now applied to the seat of pain, and the saline remedies persevered in with so much relief that he had no cough or accession of fever. The pulse became soft and regular at eighty; tongue foul and loaded. On the 12th, half past 6 A. M. reported better; no fever; slight cough, no expectoration; tongue foul; no stool; but seems getting better. Was seen by Mr. Parrock at the visit, who especially noticed this patient. 10 A. M.—For the first time, has complained of severe shooting pain all over the right thigh, particularly towards the groin, where there is slight swelling, with much heat of the parts. The vessels of the thigh appear natural and soft; states he first perceived this pain, rather suddenly, about half an hour ago, before which he was quite easy; pulse quick; skin warm; tongue clean; bowels open twice; appears rather anxious. Twelve leeches were applied to the parts affected, followed by fomentations. 12, Noon.—Leeches have just come off, and states he feels somewhat relieved; not so restless; vessels of thigh natural, but hot; distension in groin the same; pulse quick; skin warm; appears anxious. The fomentations were continued.

Half past 2 P. M.—Has become suddenly much worse; looks anxious, and nearly of a leaden hue; quick convulsive respiration, deep and almost stertorous; great oppression at præcordia and about the heart; also feeling of pain all over him, and particularly of right thigh, which is now much swollen and distended, from the hip to the knee; hard, tense, and somewhat discoloured above, but softer and elastic, and crepitating towards the knee; starts in bed at times; pulse quick and irregular, one hundred and twenty; chest sounds well, and the stethoscope reveals a curious gurgling kind of *bruit de soufflet*; but the respiratory murmur is natural, and there is no abnormal sound about the limb; pulse at groin vibrating or bounding, and no pulse in the ham. One long incision was made outside the thigh, and another near the knee, about one inch in length, which gave vent to a reddish serosity with bubbles of air.

℞. Æther. Sulph. ʒss. Tinct. Opii, m. xx. Mist. Camphor. ℥i. M. stat. sum. et repet. om. sem. hor.—Frictio.

3 P. M.—Little or no hæmorrhage from incisions, but a great deal of air escapes in bubbles, with some sanious fluid. This relieved him a little, but the restlessness continues, with short quick respiration, and he is becoming weaker and weaker, and more oppressed, and the limb more swollen, painful, and distended, and on pressure much air escapes from the incisions.—*Cont. remedia.*

Half past 3 P. M.—Pulse weaker, and a fainting sensation at times; some relief from the draught; limb the same; seems worse; more anxiety, and great distress of countenance.—*Cont. remedia.*

Quarter to 4 P. M.—Sinking fast; cold clammy sweats; respiration more laborious; pulse nearly gone; still air in bubbles come away from the incisions.—*Cont. remed.*

Died five minutes to 4 P. M.

*Sectio Cadaveris* one hour and a quarter after death, in the presence of Messrs. Smith, Kennedy, Coledridge, and Parrock.

*Head.*—On removing the *dura* and *pia mater*, about six ounces of serous fluid escaped; a few bubbles of air were observed on the surface of the *pia mater*. The brain was softer than natural; about three drachms of pale serous fluid were contained in each lateral ventricle.

*Thorax.*—Left lung collapsed; the right lung had old adhesions to *pleura costalis*, and both lungs appeared healthy. About two ounces of serous fluid were contained in pericardium, and some air bubbles, like vesicles, on its surface. The heart was pale, flabby, and collapsed, with several small bubbles of air on its upper surface, the size of millet-seed. The heart was carefully removed, and the vessels compressed, in order to exclude the external air, and immersed in water, when some air rose to the surface from the right side. This viscus was found nearly empty, and contained sufficient blood only to tinge the fluid of a light red colour.

*Abdomen.*—Liver rather larger than natural, more brittle in texture, and of a pale dram-drinking appearance. Great hypertrophy of the spleen, which had increased to nearly twice its natural size, and was of a dark purple colour. A few ulcerated spots were observed in the large intestines. The mesenteric glands were enlarged. The other viscera were healthy.

The *right thigh* was much enlarged, hard, distended, shining, and somewhat discoloured; when punctured giving out much gas. On turning back the skin and fasciæ, the muscles were seen of a dark purple colour, commencing about the origin, and extending half-way down the thigh; and when incisions were made into them, a quantity of air and fluid blood escaped. The blood and air seemed to be pretty equally diffused throughout the whole tissues of the muscles, appearing like clotted blood, giving a crepitating feel like lung, but appeared to be more abundant in the extensor and abductor muscles. The Sartorius muscle free from disease, but much swollen. The blood-vessels were healthy. The femoral artery and vein, with their large branches, were carefully dissected. The cellular tissue of the large blood-vessels was infiltrated with dark blood, and bubbles of air accompanying the vessels under Poupart's ligament to the fore part of the thigh. The femoral vein contained some air; all the other vessels containing merely fluid blood. Much air issued, with frothy sanious fluid, when cut into and exposed. It appeared to have transuded and given out the air or gas.

The remarkable features of this case during life, were its sudden and unaccountable appearance; the rapid sinking and great distress in the course of its brief career; the sudden swelling, and crepitating feel of the limb; the issue of bubbles of air, with a watery serosity, when the distended parts were incised; the leaden hue and great anxiety of countenance, &c. The important circumstances disclosed by dissection were bubbles of air on the surface of the *pia mater*; the softening of the brain; air bubbles, like vesicles, on the pericardium; the heart pale, flabby, and collapsed, with air globules on its surface, and air contained in the right side of the heart; the enlargement of the diseased limb giving out gas when punctured; the dark purple colour of the muscles also containing air and fluid, and its diffusion through the connecting tissues of the muscles, giving them the appearance of hepatized lung, as well as crepitating; the cellular tissue of the large vessels infiltrated with dark fluid blood, and vesicles of air; the femoral vein containing gas, but the other veins, fluid blood, and their tissues, a frothy sanious fluid, which appeared to have transuded, and given out air or gas. The femoral vein and diseased muscles were removed, washed, and then put into water. When examined twelve hours after, they were increased in bulk, and covered with bubbles of air, and still gave the crepitating feel of lung. The small quantity of air preserved, a part had a light put to it without any very apparent result; the other portion with lime-water indicated carbonic acid gas.

Did the extrication of the gas arise from secretion or spontaneous evolution? or from partial decomposition or alteration of the circulating fluid? Its local origin, and total unconnection with respiration, and altered state of the blood, as if deprived of its vitality, would countenance the latter supposition, arising, perhaps, from this morbid state of the vascular action, connected with depressed vital powers, giving rise to its apparent secretion, and, had the patient lived long enough, might have become a case of humid gangrene. The blood had lost its property of coagulating, giving rise probably to its tendency of extricating gas, as it seemed to transude with bubbles of air, which continued even after dissolution.

The immediate cause of death may have arisen from air getting into the circulation, as the heart was pale, flaccid, and flabby, and had air in its right cavities.

*Bangalore, East Indies, June,* 1839.

*Ibid.*

---

*On the Injurious Qualities of Menstrual Blood, and on its Probable Causes.* By Dr. Remak, of Berlin.—This paper is chiefly useful for the information it affords respecting the composition of a fluid which few have either the inclination or the liberty to examine. The author adduces but little fresh evidence in support of the universal popular belief that menstrual blood can produce urethral discharge or other affections of the male genital organs; but he thinks that the occasional occurrence of these symptoms, of which he has no doubt, depends on the condition, not of the blood, but of the mucus with which it is mixed.

By microscopical examination of the menstrual fluid under various circumstances, he found that whenever it had a red colour, it contained blood-globules, and that the intensity of its colour depended on their number. At the beginning and towards the end of its flowing, when it is pale and whitish, it contains a preponderating quantity of laminæ of epithelium and of mucus-corpuscles, but their quantity becomes proportionally less and less as the fluid presents more blood-globules and a deeper colour. The author believes that this mucus which is constantly present in the menstrual fluid, may, like the secretions of all mucous membranes when in a morbid state, possess the power of producing a similar morbid state in other membranes of the same kind. He relates a case which confirms this idea: a very obstinate gonorrhœa, in which the menstrual fluid of the only female with whom the patient had connection, and in whom there was not the least probability of gonorrhœa existing, contained an abundance of thin pearl-coloured mucus. Connection did not produce the gonorrhœal symptoms, except when the female was menstruating.—*Brit. and For. Med. Rev., from Medicinische Zeitung.* Dec. 25, 1839.

---

*Evolution of Heat in Plants.* By MM. Vrolik and Vriese.—Some additional experiments on *colocasia odora* have been recently made by MM. Vrolik and Vriese. Besides affording a complete confirmation of the results obtained by others, they have established some new and important facts. A flowering spadix placed in oxygen, was compared with a corresponding one in atmospheric air. Within half an hour after its immersion, its temperature rose to 2·7° C. (4·9° F.,) whilst that of the one in air was falling 1·1° C. (2° F.) During and after the emission of the pollen in both, the maximum temperatures were the following:

| | | | |
|---|---|---|---|
| Spadix in Oxygen | . | 30·6° C. | 87·1° F. |
| Surrounding Temp. | . | 23·3° C. | 74·0° F. |
| Difference | . . | 7·3° C. | 13·1° F. |
| Spadix in Atm. Air | . | 27·8° C. | 82° F. |
| Surrounding Temp. | . | 22·8° C. | 73° F. |
| Difference | . . | 5·0° C. | 9° F. |

The temperature of the spadix in oxygen rose much more rapidly than that of the other; so that early in the day the difference was 4·6° C.; that of the surrounding media being ·3° in favour of the lowest, so as to make the difference really 4·9° C., or 8·8° F., whilst that of the maxima of both was 2·3° C., or 4·1° F. In these experiments, a large quantity of oxygen was observed to disappear; on the other hand carbonic acid was liberated, which, being absorbed into the water below, caused it to rise into the vessel.

A spadix beginning to flower was placed in nitrogen. It had already acquired an elevation of temperature to the amount of 2·6° C. This gradually diminished, although the expansion of the flower continued. At the time of the emission of the pollen there was no elevation; whilst a corresponding spadix in the open air showed an elevation of 8·6° C. No change in the bulk of the confined gas was noticed in this experiment.

These experiments afford a striking confirmation of the doctrine now rapidly gaining ground amongst physiologists, that the evolution of caloric from living beings is dependent upon certain organic processes, in which the combination of oxygen (either immediately or remotely derived from without) and the carbon of the system are principally concerned. These go on most actively during the period of inflorescence, but they are not restricted to it. The same process of *respiration* is constantly going on in almost every part of the living plant; but, owing to its feeble amount, and the extended surfaces by which the caloric is dissipated as fast as it is generated, it is difficult to acquire any evidence of its liberation.

By the use of the thermo-multiplier, however, which was so successfully employed by MM. Becquerel and Breschet in their experiments upon animal temperature, M. Dutrochet has succeeded in proving the existence of a *proper heat* in plants, more especially in the green parts, which are the seat of the more active organic changes. In order to obtain unequivocal evidence to this effect, it was necessary to devise means of excluding the other causes which produce an elevation of temperature in some parts of the vegetable structure. Thus, in a growing tree, the sap which rises in spring, being principally drawn from a depth of two or three feet in the soil, will be warmer, perhaps even by several degrees, than the atmosphere. On the other hand, the evaporation constantly going on from the soft and moist surfaces of rapidly-growing parts, will depress their temperature. M. Dutrochet employed in his experiments plants either growing in pots, or cut off and placed in water of the same temperature as the surrounding air; and he did not rely upon the temperature of the stem, but upon that of the leaves and young shoots, in reaching which the sap would have acquired the temperature of the plant. He thus avoided the first source of error. The second was prevented by making his comparison, not between these parts in a growing plant and the surrounding atmosphere, but between corresponding parts in a living plant, and in one recently killed by immersion in hot water, and therefore susceptible of the diminution of temperature which evaporation causes, to the same degree as the living plant. The experiment was in some instances still further prevented from error by the immersion of both plants in an atmosphere saturated with aqueous vapour. With these precautions the result was *constantly* the same. An elevation of temperature to the amount of one-fourth or even one-third of a degree (Cent.) was observed in the herbaceous parts of actively-growing plants, differing with the species, the activity of vegetation, and the period of the day. There is a sort of diurnal paroxysm, which generally manifests itself about noon; in some species an hour before, in others an hour after. The *proper heat* increases up to this period, attains its maximum, and then diminishes. The access of this paroxysm depends in part upon the presence of light; but it will occur, though in a diminished degree, for two or three days in a plant withdrawn from the influence of that agent.—*Ibid., from Annales des Sciences Naturelles*, N. S. Botan. Tom. xi., xii.

---

*Experiments on the Motor and Sensitive Roots of the Nerves.* By Dr. KRONENBERG, of Moscow.—During my stay in Paris, in July, 1839, Prof. Magendie had the kindness to show me his recent experiments on the nerves, which he had communicated to the Academy. These, as made in my presence, I will here detail, in the first place, and then notice my own experiments made some time afterwards.

1. M. Magendie laid bare the facial nerve of a dog near its origin, and pricked it; at each prick a demonstration of pain was given. The nerve was then cut across, not far from its junction with the fifth pair. Pricking and pinching the cerebral end of the divided nerve produced no indication of sensibility; but pinching the other end obviously caused pain. It appears from this experiment that the sensibility of the facial nerve, as well after as before its union with the fifth pair, depends on this last, and not from any other anastomosis or the influence of the brain sent direct through its origin. M. Magendie then laid bare the roots of the lumbar nerves of a dog. The posterior roots were found as usual very sensible; the anterior were less so; still the dog whined and gave evident signs of pain each time they were pinched. M. M. then divided the motor root; pinching the spinal extremity of this produced no pain, but pinching the other (before its union with the sensitive root) caused the dog to cry out.

2. Some weeks after this I performed the following experiments on rabbits:—The facial nerve before its union with the fifth was found sensible, at one time more so, at another less; on dividing it before its union, pinching its under (peripheral) end produced pain, yet not always. I then laid bare the lumbar portion of the spinal marrow, and found the motor roots sensible, but much less so than the sensitive. The following experiments, however, prove that the sensibility of the motor roots is not derived through fibres coming directly from the spinal marrow, but is dependent on the other (the sensitive) roots. When I stimulated the motor root, the sensitive root being undivided, pain was evinced; but when the latter was divided, the same stimulus produced no sensation. Magendie's experiment equally proved this, and its repetition by myself confirmed the same. After division of the anterior root, the posterior being untouched, the under or peripheral end was always sensible, the upper not. The case is similar with the anterior column of the spinal marrow—pain being only produced when the posterior roots were uninjured.

In order to settle this point still more firmly, and to ascertain the course of the fibres, I made the following experiment:—I made in the angle of union of the two roots a small incision, about half a line in extent, leaving both the roots untouched: it was then found that the same phenomenon no longer took place; thus, the anterior root and the anterior column of the spinal marrow were now insensible, and on the division of the root both its ends were equally insensible.

This simple and easy experiment proves, first, that a portion of the fibres of the sensitive root extends to the point of union and is reflected back to the anterior column of the spinal marrow; and, secondly, that the return or reflection of the fibres takes place near the point of junction of the two roots.

[These experiments afford a satisfactory explanation of phenomena which have been very perplexing to physiologists. It has been almost constantly observed that some degree of sensibility appeared to exist in what Sir C. Bell regarded as exclusively the *motor* roots of the nerves; this sensibility being manifested by expressions of pain on the part of the animal when they were irritated. The sensibility of the portio dura has long been known, and was correctly attributed by Sir C. Bell to its reception of filaments from the fifth pair. A similar mixture of the filaments of the posterior roots of the spinal nerves with those of the anterior—these filaments passing *towards* the centre as well as *from* it—may be reasonably anticipated; and this supposition fully explains all the phenomena which have led Magendie and others to the opinion that the anterior roots are sensible.]—*Ib., from Müller's Archiv.* Heft. v., 1839.

---

*On the Nerves of the Cornea.* By Dr. PAPPENHEIM.—The cornea has been represented by M. Hippolyte Cloquet to be devoid of nerves as well as of blood-vessels. In 1830, however, Schlemm succeeded in tracing nerves as far as the margin of the cornea, though its density prevented his following them between its laminæ. The investigation thus commenced has been pursued by M. Pappenheim, who, by a simple process, that of immersing the cornea in acetic acid, or a solution of caustic potass, has been able to trace nerves from the sclerotica into the substance of the cornea. That the nerves thus traced really belong to the cornea, the following facts appear to prove:—1. If the corneal conjunctiva is removed, the nervous filaments are seen on the inner, not on the outer surface of the corneal epithelium. 2. The removal of the iris and membrane of the aqueous humour makes no difference as to the ease with which the nerves may be seen. 3. The nerves are distinctly visible entering the margin of the cornea, but less so towards its centre, where they are ultimately lost between the laminæ.

The nerves may be distinguished from the folds of the choroid which mark the cornea by being colourless, and of uniform thickness; from the fibres of the cornea by being smaller, more superficial, scattered, and arranged in plexuses. Internally they are covered by the membrane of the aqueous humour, externally by the fibres of the cornea. The readiest way of discovering them is to immerse the dissected cornea in water, placing it between two plates of glass, with its inner surface turned upwards; gentle pressure, and the light of a lamp are required, and at first a slightly magnifying lens will be desirable. The nerves will then be seen taking the course of the blood-vessels, and composed of fasciculi for the most part separate.—*Ibid., from Monatschrift für Medicin,* &c. June, 1839.

---

*Adhesions of one of the Aortic Valves to the surface of the Aorta, and obliteration of one of the Coronary Arteries.*—Dr. Kingston showed, at the Pathological meeting of the London Royal Medical and Chirurgical Society, a preparation exhibiting two lesions, no cases of either of which have yet been recorded; one of these was a perfectly close adhesion of one of the aortic valves through its whole extent to the surface of the aorta. In addition to tough, thready membrane, connecting the surface of the valve to the artery, there was a thin, firm, reddish membrane passing from the aorta straight over the free edge of the valve, a minute portion only of which it left uncovered. This membrane extended for about an inch upon the surface of the ventricle, its extremity being in some places loose and shreddy; the adherent valve, as well as the other two, had a little cartilaginous thickening at its edges; the portion of the aorta to which it adhered was atheromatous, and greatly thickened, and the rest of the upper part of the aorta was irregularly thickened with patches of atheroma, under the inner membrane, alternating with cartilaginous degeneration of the inner membrane itself; the orifice and channel of the aorta were of moderate calibre.

The other remarkable lesion exhibited by this preparation was a total obliteration of the orifice of the left coronary artery, and thence of its channel for the distance of an inch; this part of the channel was flattened, with firm adhesion of its opposite surfaces; the right coronary was of rather small calibre, and healthy texture.

With respect to the heart, the tricuspid orifice was dilated to a circumference of five inches, while its valve was somewhat shortened; the cavities were all greatly dilated, but those of the left side much the most so; the left ventritricle was attenuated nearly in proportion to its dilatation; the right ventricle was somewhat hypertrophous.

There was extensive bronchitis, pulmonary apoplexy, rather recent adhesion of the left lung to the pericardium, granular liver, the mucous membrane of the stomach deeply reddened, and softened over its whole extent.

The subject of these lesions had been a married woman, aged fifty-three; she had, in the last stage of her illness, been a patient of Dr. Kingston and Mr. Walsh; in youth, but not of late years, she had been subject to articular rheumatism; her fatal complaints had come on four or five years ago at the decline of the catamenia; she used to be seized, while walking, with urgent dyspnœa, pain extending from the heart to the left scapula, and such extreme faintness as to be in danger of falling; she used frequently to wake in the night with similar sensations, obliging her to start up in bed, and continue erect for a considerable time; there was great weight at the stomach after food, frequently combined with crampish pain and vomiting. In both kinds of paroxysm she derived relief from hot stimulating drinks.

Six months before death her complaints were much aggravated by affliction at the death of her husband, during the last night of whose life she was in a state of alarming syncope for some hours. Cough supervened, with great excitability, depression of spirits, and debility; the pulse used to be about a hundred while in bed, but was much accelerated, and sometimes unequal after the exertion of walking; it was not decidedly deficient in firmness and fulness; there was none of that visible pulsation of the arteries which has been supposed pathognomic of aortic regurgitation; there was a rough, sawing murmur in the region of the ventricles, owing to the regurgitation at the tricuspid orifice; and there was a strong blowing murmur at the region of the semilunar valves, owing to the regurgitation through the aortic orifice, one-third of which was permanently patent.

On admission to the dispensary, fourteen weeks before death, she obtained great relief for six weeks from tonics, antispasmodics, and carminatives; but she relapsed, and hydropericardium and anasarca supervened; she was confined to bed a month, and sunk slowly.

In connection with this case Dr. Kingston mentioned another he had met with, in which the orifice of one of the coronary arteries, though not quite obliterated, was reduced to the breadth of a small pin; it was surrounded by a yellow rim; the channel beyond was much contracted, and drawn up into longitudinal folds for the distance of three quarters of an inch; the other coronary artery was much narrowed in various parts of its course by atheromatous and osseous thickening; there was a little cartilaginous thickening of the aortic valves, an atrophic perforation of the mitral valve, great thickening, partly cartilaginous and atheromatous, of the aorta, great hypertrophy of the left ventricle, combined with great dilatation of all these orifices and cavities.

The subject had been a sweep, aged forty-eight; had occasionally had rheumatism of no great severity; had for ten years been subject to vertigo, dsypnœa on exertion, and wheezing; and for five or six years to cardiac palpitation. About four months before death the dyspnœa greatly increased, and was conjoined with a severe epigastric pain, "as if he were drawn up in knots," on walking fast or lying down, and often with a sensation as of something rising to the larynx, and producing sense of suffocation, which often waked him in the night, and obliged him to start up and walk about; the pulse ranged from one hundred and four to one hundred and twenty-four, and was harsh, hard, firm, moderately full; the heart's impulse was very strong, and extended over and to the left of the cardiac region; never any œdema; what most relieved him was a small bleeding, and an antimonial saline mixture.

The day before death he was easier, and more cheerful than previously. At night, after being in bed some hours, he got out, saying, "I am very ill!" After walking about the room for a minute he returned to bed. Five minutes afterwards he jumped up, exclaiming, "I am dying!" and, after one or two gurglings in the throat, fell down dead.—*London Lancet.*

